# Predicting hepatocellular carcinoma through cross-talk genes identified by risk pathways

**DOI:** 10.18632/oncotarget.24915

**Published:** 2018-04-20

**Authors:** Lei Liu, Lin Pang, Yunfeng Wang, Ming Hu, Zhuo Shao, Diwei Huo, Denan Zhang, Hongbo Xie, Jingbo Yang, Qiuqi Liu, Xiujie Chen

**Affiliations:** ^1^ College of Bioinformatics Science and Technology, Harbin Medical University, Harbin, Heilongjiang Province, China; ^2^ The 2nd Affiliated Hospital of Harbin Medical University, Harbin Medical University, Harbin, Heilongjiang Province, China

**Keywords:** cross-talk genes, risk pathways, molecular markers, hepatocellular carcinoma (HCC)

## Abstract

Hepatocellular carcinoma (HCC) is the most frequent type of liver cancer with poor survival rate and high mortality. Despite efforts on the mechanism of HCC, new molecular markers are needed for exact diagnosis, evaluation and treatment. Here, we combined transcriptome of HCC with networks and pathways to identify reliable molecular markers. Through integrating 249 differentially expressed genes with syncretic protein interaction networks, we constructed a HCC-specific network, from which we further extracted 480 pivotal genes. Based on the cross-talk between the enriched pathways of the pivotal genes, we finally identified a HCC signature of 45 genes, which could accurately distinguish HCC patients with normal individuals and reveal the prognosis of HCC patients. Among these 45 genes, 15 showed dysregulated expression patterns and a part have been reported to be associated with HCC and/or other cancers. These findings suggested that our identified 45 gene signature could be potential and valuable molecular markers for diagnosis and evaluation of HCC.

## INTRODUCTION

Liver cancer, with an increasing incidence rate, is the second most frequent cause of cancer related death worldwide [1]. The most frequent type of liver cancer is hepatocellular carcinoma (HCC), which originates from the main liver cells and accounts for 70%–85% of primary liver cancer, with poor 5-year survival rate and high mortality.

Many factors can cause the liver cirrhosis which increases the risk of HCC, including chronic hepatitis B or C infections, heavy and prolonged alcohol consumption, diabetes and obesity and some inherited liver diseases. Hepatocellular carcinoma is usually diagnosed by computed tomography or magnetic resonance imaging, followed by a liver biopsy to confirm the diagnosis.

Previous studies have identified many drivers with frequent mutations and aberrant expressions in HCC, such as TERT, TP53, CTNNB1 and ARIDA1A [2, 3]. Although further analyses explored the mechanism of tumorgenesis mediated by these drivers, the understanding of liver cancer remains to improve and deepen. Therefore, novel molecular markers are still urgently needed, which benefits for early diagnosis and risk assessment.

Moreover, hepatocarcinogenesis is a complex multistep process with multiple signaling cascades changed, including vascular endothelial growth factor (VEGF) signaling [4], Ras MAPK signaling [5], the PI3K/PTEN/Akt/mTOR pathway [6] and Wnt/β-Catenin pathway [7]. Notably, interactions between signaling pathways frequently occur and have been elucidated in many cancers, which emphasizes the roles of cross-talk between key pathways or regulators in the genesis and development of tumor. For example, Lihong Xu and colleagues found that the cross-talk between the PPARδ and prostaglandin (PG) signaling pathways could contribute to the hepatocarcinogenesis through regulating HCC cell growth [8]. The cross-talk between the PI3K/Akt and MEK/ERK cascades could result in cell cycle arrest and cell survival in order to adapt to endoplasmic reticulum (ER) stress [9]. Some studies also focused on the cross-talk genes. For example, Jiang Du found that the dys-regulated genes in ARLC-SCC enriched for many pathways which showed obvious correlations by sharing cross-talk genes. They further identified 8 cross-talk genes which bridge multiple ARLC-SCC-specific pathways as candidate biomarkers [10]. Kang Ae Lee performed a comprehensive analysis to determine the extent of cross-talk between the AHR and HIFs and focused on the 33 shared genes between the two sets of genes exhibiting sensitivity to cross-talk [11]. These findings suggest that, investigating cross-talk between cancer-related pathways and genes can promote our understanding of the mechanism of tumorigenesis.

Here, we used expression profiles of HCC to identify markers which could accurately distinguish patients and healthy people, as well as the prognosis of HCC patients, through integrating network and pathway information with our developed method. These markers provide valuable choices for diagnosis, targets for treatment and objects for further analysis of HCC.

## RESULTS

### Identification of differentially expressed genes

**Figure 1:**
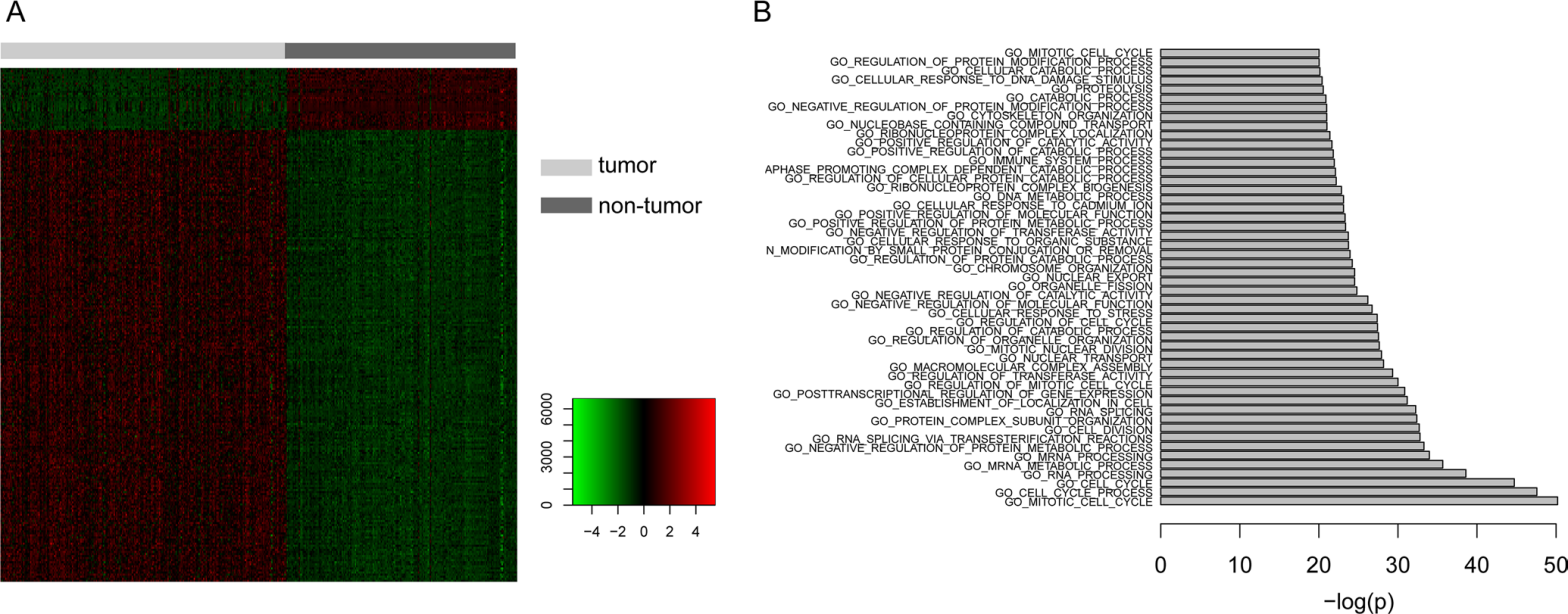
The analysis of differentially expressed genes (**A**) Heatmap showing the 249 significantly differentially expressed genes, consisting of 219 up-regulated genes and 30 down-regulated genes. (**B**) Functional enrichment analysis of the differentially expressed genes with x axis represents the negative log10-transformed *P* values.

In order to identify dysregulated genes during the genesis of HCC, we first obtained the microarray expression profile of a total of 433 samples including cancerous and pericarcinomatous tissues of HCC. Using one of the widely used method Limma [12], we identified 249 significantly differentially expressed genes, consisting of 219 up-regulated genes and 30 down-regulated genes (Figure 1A, Supplementary Table 1). Further functional enrichment analysis demonstrated that these dysregulated genes were involved in many cancer-associated biological processes, such as metabolism [13], immune system [14] and especially the cell cycle [15] (Figure 1B), suggesting the dominating abnormity of cell proliferation in HCC.

### The construction of HCC-specific interaction network

**Figure 2:**
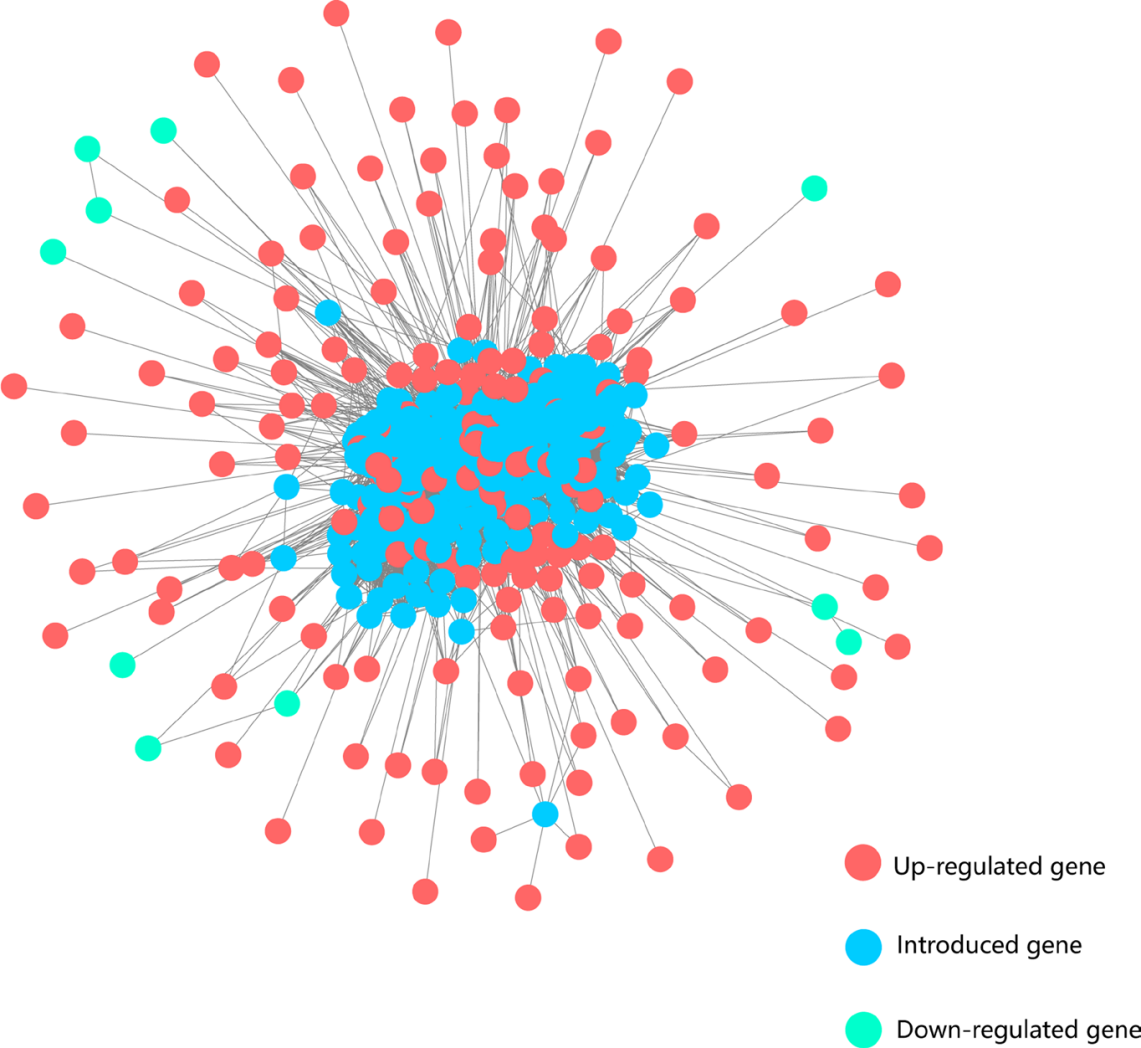
The HCC-specific network Red and green nodes represent up-regulated and down-regulated genes, respectively. Blue nodes represent the introduced genes which connect directly to the differentially expressed genes in the background network.

Given that a biological process is synergistically regulated by many factors other than independently by one gene, we assumed that the dysregulated genes controlled liver-associated physiological activities through interacting with each other or additional regulators. To obtain the dysregulated network of HCC, we mapped the 249 differentially expressed genes to the protein interaction network consisting of 14553 protein-coding genes (PCGs) and 662360 interactions between them, which was built by combining resources from BioGrid and HPRD database. Based on this integrated network, we extracted a HCC-specific network which contained differentially expressed genes and their closely associated PCGs (see Method). Finally, the HCC-specific network was composed of 522 nodes and 12841 edges (Figure 2). Notably, many genes with large degrees in this subnetwork have been reported to be associated with HCC, such as UBC, SUMO2, SNW1, POLR2A, CDC5L, CDK1, PLK1 and HNRNPK. Functional enrichment analysis showed that, compared with the dysregulated genes, genes of HCC-specific network additionally enriched for processes about cell cycle phase transition, innate immune response and NF-kappaB signaling, which were not captured by the dys-regulated genes but were associated with cancer process (Supplementary Table 2). These findings suggested that the subnetwork may contain key regulators of liver pathophysiology, whose aberration may play important roles in HCC mechanism.

### The identification of important genes based on HCC-specific network

Considering the importance and biological significance of hub genes in the interaction network and the close relationship of the subnetwork with HCC, we calculated a state score for each gene that combined the extent of the deviation and the degree in the HCC-specific network to characterize its transcription status and importance (see Method). Totally, we identified 480 genes which contained 455 up-regulated genes with state scores being positive and 25 down-regulated genes with state scores being negative, for subsequent analysis (Supplementary Table 3).

### Risk pathways can distinguish disease and normal samples

The dysregulation of crucial pathways commonly occur in cancer, which were frequently caused by the aberrant activation or repression of key genes. Therefore, we used DAVID to obtain the enriched pathways of 455 up-regulated genes and 25 down-regulated genes identified above, respectively. Totally, we obtained 31 pathways, among which 9 pathways contained both up-regulated and down-regulated genes, 18 pathways only contained up-regulated genes and 4 pathways only contained down-regulated genes.

**Figure 3:**
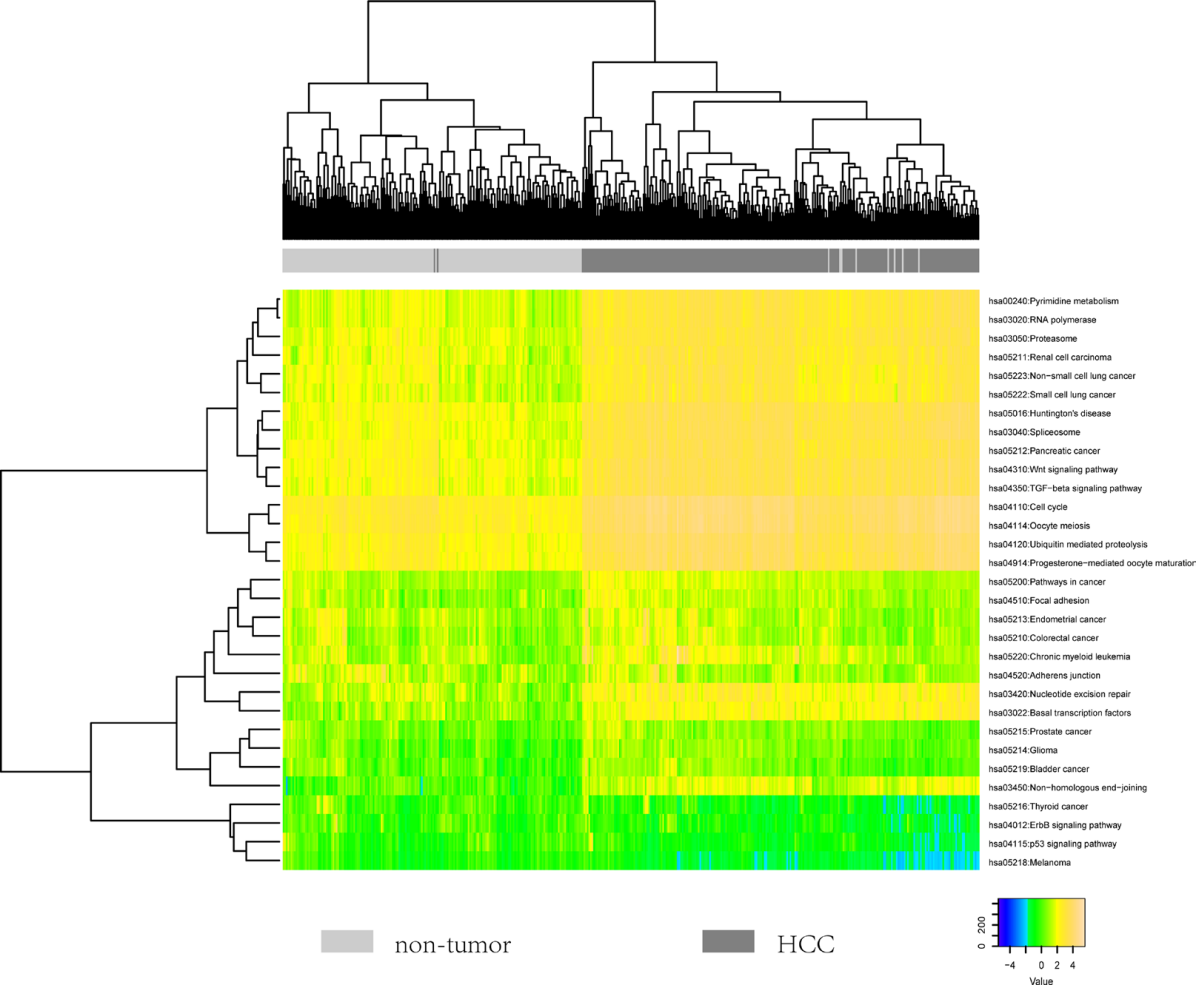
The heatmap showing the hierarchical clustering of all samples using the scores of 31 identified pathways

To explore whether these pathways could reflect disease status, we calculated a score for each pathway based on the expression profiles of up-regulated and down-regulated genes. Hierarchical clustering analysis of the 31 pathways based on our defined scores could nearly completely distinguish HCC and normal samples (Figure 3). Notably, pathways such as Wnt signaling pathway, cell cycle and TGF-beta signaling pathway showed active status. On the contrary, we found that focal adhesion and adhesions junction showed inactive status, which implied the tendency of metastasis. Moreover, we observed that many other cancer-associated pathways were dysfunctional. These results suggested that these pathways could reveal the highlighted cancer-associated changes during the pathogenesis of HCC.

### The identification of cross-talk genes and their prediction efficiency

The biological regulation network is a complex system, in which the interactions between pathways are common and important to maintain many pivotal biological processes. The cross-talk genes are shared by pathways and are considered as indispensable members whose dysfunction may result in serious consequences including diseases. To further explore which caused the variety between HCC and control samples, we focused on the cross-talk genes between the 31 pathways identified above. Of the 223 gene in all these pathways, we found that 104 genes were shared by at least two pathways, which we considered as cross-talk genes and were used for subsequent analysis (Supplementary Table 4). For example, MAPK1, TP53, EGFR and RB1 were well-known HCC-associated genes whose alteration were frequently observed in multiple layers including genome, transcriptome and epigenome. Moreover, we also found some other cross-talk genes which had few reports about HCC, like some subunits of the anaphase-promoting complex/cyclosome (APC/C) complex (ANAPC1, ANAPC2, ANAPC4, ANAPC5, ANAPC7, ANAPC10 and ANAPC11). Moreover, the pathways enriched by the 104 cross-talk genes and found that they were involved in many cancer-associated biological processes, such as TGF-beta signaling pathway, Wnt signaling pathway, adherens junction and cell cycle, implying their potential roles in HCC.

**Figure 4:**
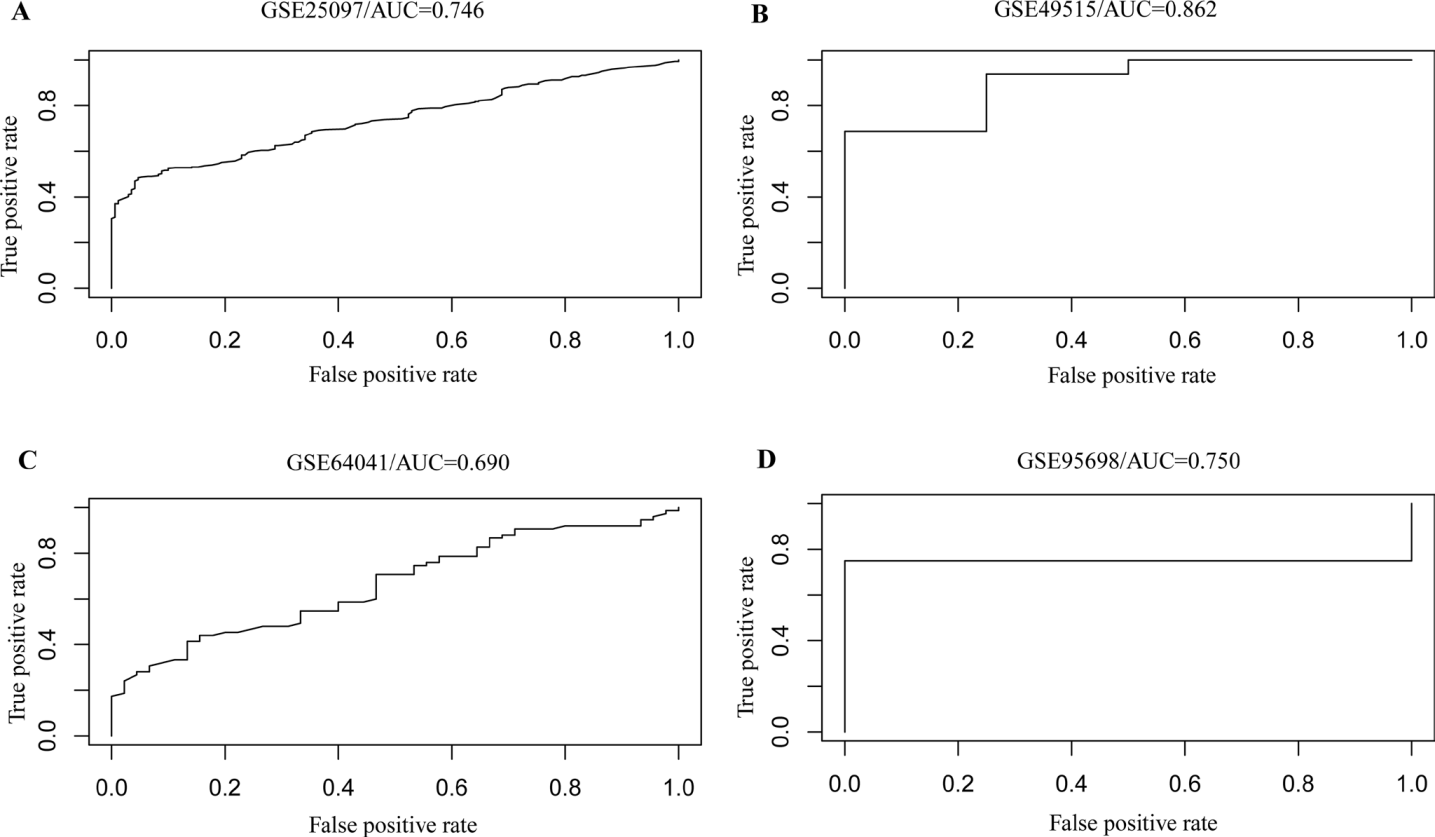
The ROC curve of our model showing its power of distinguishing HCC patients with normal individuals in additional two array data (**A** and **B**) and two RNA-seq data (**C** and **D**).

To extract reprehensive genes from the 104 cross-talk genes, we used Weka [16] to carry out feature selection and further identified 45 genes which could reflect more information of samples and disease status. Then we constructed a model using random forest algorithm based on the expression levels of these 45 genes in the 433 samples. Notably, these 45 genes contained 15 differentially expressed genes. All these 45 cross-talk model genes were found closely related to cancer (Supplementary Table 5 and Supplementary Table 6). To detect the accuracy of our model, we obtained another two microarray data sets and two RNA-seq data sets. We applied our model to these four data sets and found it could accurately distinguish HCC patients and normal individuals. The AUC were 0.746 and 0.862 for the two expression profile data sets, as while as 0.690 and 0.750 for the two RNA-seq data sets, respectively (Figure 4). Further, permutation analysis validated the significance of this model in the four data sets with *P* value of 0.05 (see Method).Taken together, these findings suggested that the 45 cross-talk genes we identified could be valuable biomarkers that were of clinical significance for diagnosis of HCC.

**Figure 5:**
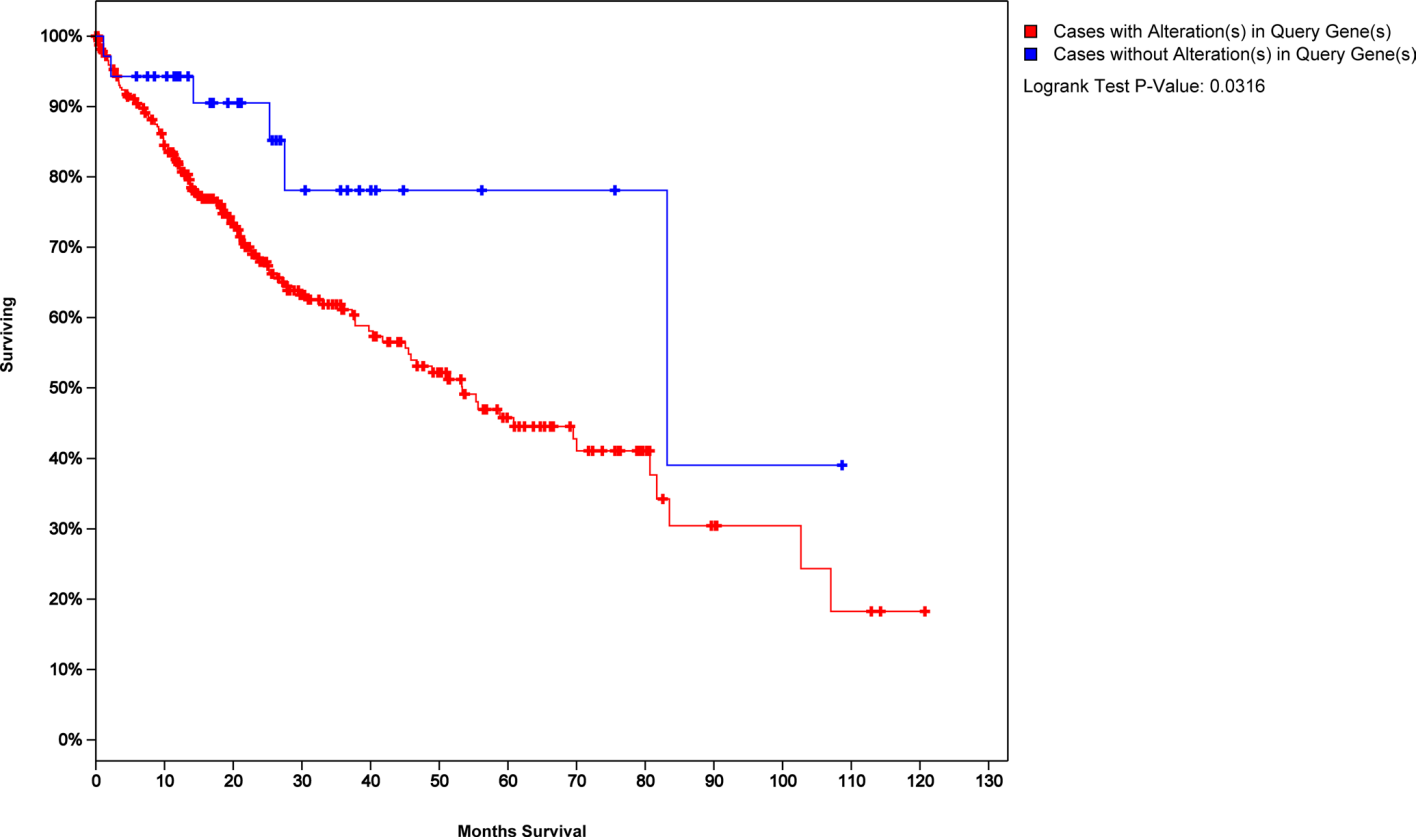
Survival analysis of HCC patients with 45 cross-talk model genes as a tag

As our model was constructed with cross-talk genes which might reveal more mechanism with HCC, we wondered if they could reveal the prognosis of HCC patients. We used the 45 cross-talk model genes as a tag and made a survival analysis of HCC patients (Figure 5, see Method). The blue curve represented the survival time of HCC patients with 45 cross-talk model genes not exhibiting changes and the red curve represented survival time of HCC patients exhibiting changes in at least one of these 45 cross-talk genes. A *P* value of 0.03 indicated that the 45 cross-talk genes may also serve as biomarkers to assess the prognosis of HCC patients.

## DISCUSSION

Hepatocellular carcinoma severely influences the quality of patients’ life, which promotes us to develop new and effective markers for diagnosis and treatment of HCC. In this paper, we integrated expression, networks and pathways to identify valuable markers of HCC. The 45 gene signature we identified could accurately distinguish HCC patients and normal individuals, implying their potential clinical application.

The liver can regenerate after either surgical removal or after chemical injury. It is known that as little as 25% of the original liver mass can regenerate back to its full size [17]. Consistently, the differentially expressed genes mainly enriched in cell cycle-associated processes, such as mitotic cell cycle, cell cycle process and cell division, demonstrating that these dysfunctional genes could really reflect the physiological and pathological characteristics of HCC.

Almost all of the biological processes were precisely regulated by a diversity of factors, which make up a complicated network. Here, we extracted a subnetwork which contained many HCC-associated genes, such as UBC, SUMO2, SNW1, POLR2A, CDC5L, CDK1, PLK1 and HNRNPK. Interestingly, UBC and SUMO2 were well-known ubiquitin-associated genes, which have been reported to be involved in cell-cycle process [18] and DNA damage [19, 20]. The knockdowns of the spliceosome protein SNW1 could result in mitotic arrest [21]. Since RNA polymerase II mediates the transcription of all protein-coding genes in eukaryotic cells, POLR2A, as the catalytic subunit of it, was frequently detected to acquire mutations in cancers [22], suggesting its important roles in the cancer development. Moreover, many studies have found that CDC5L, CDK1 and PLK1 were crucial regulators of cytokinesis [23, 24]. All these findings were concordant with the results above, suggesting that cell cycle and cytokinesis may be the major disturbed processes during the genesis of HCC.

During the cancer development, many key pathways showed aberrant changes. Notably, interactions between pathways are also of significance for normal life activities, the destruction of which may cause severe diseases including cancer. The cross-talk genes are the ones shared by pathways, which are considered as key regulators in biological processes. Based on this assumption, we finally identified 45 cross-talk genes, selected from the 31 risk pathway we identified through combining the defined up- and down-regulated genes. We assumed that these cross-talk genes could reflect more disease-associated information since they participate in different pathway regulation simultaneously and help us to accurately characterize the features of patients with HCC. Consistently, applying these 45 genes to additional data sets showed that they could distinguish HCC patients and normal individuals with a high precision. Moreover, among the 45 genes, some genes has been considered as HCC biomarkers, such as SUMO2, PLK1, CCND1, CDK2 and RB1 [25, 26], suggesting that our method could capture the key changes of transcriptome in HCC development. Taken together, our work provided valuable sources of HCC-associated biomarkers which could be the alternative objects for future studies to further explore the mechanism and extend our understanding of HCC, which are all benefit for the clinical diagnosis and treatment of HCC.

## MATERIALS AND METHODS

### Data acquirement

The expression profile data (GSE36376) of hepatocellular carcinoma, which was used for identification of molecular biomarkers, contains 240 patient samples and 193 control samples.

We used two expression profile data sets and two RNA-seq data sets for validation of the prediction efficiency of molecular biomarkers. The two expression profile data sets were GSE25097, which contained 268 HCC tumor and 243 adjacent non-tumor samples, and GSE49515, which contained 10 HCC tumor and 10 adjacent non-tumor samples. The two RNA-seq data sets were GSE64041, which contained 60 HCC tumor and 60 adjacent non-tumor samples, and GSE95698, which contained 3 HCC tumor and 3 adjacent non-tumor samples. An independent dataset containing mRNA data and clinical information of 370 HCC patients from The Cancer Genome Atlas (TCGA-LIHC, https://cancergenome.nih.gov/) was used to analyze survival time. The cBioPortal database was used to generate a K-M Survival curve using cross-talk model genes [27].

All these data were downloaded from GEO database (http://www.ncbi.nlm.nih.gov/geo/). All probe expression profile data were converted to gene symbol according to their platforms information.

### Identify differentially expressed genes

We used the scale function of R to standardize the expression profiles. The differential expression analysis between tumor and control samples were carried out by the R package Limma [12]. Limma is one of the most commonly used statistical methods for analysis of differential expression, which is based on the empirical Bayes linear modeling approach. Genes with the *P* value < 0.05 and absolute fold change > 1.5 were considered as differentially expressed.

### The construction of HCC-specific network

First, we downloaded the interaction pairs of genes from BioGrid [28] and HPRD [29] database, respectively. Then, the union of these two sets of interaction pairs was used to construct a background network. Second, we mapped the 249 differentially expressed genes to the background network and extracted the genes which had at least five direct interactions with differentially expressed genes. Finally, these genes and differentially expressed genes made up a subnetwork, which was called the HCC-specific network.

### The state score

To further identify important genes, we determined a state score (W) for each gene in the HCC-specific network, which combined the extent of the deviation (E) and the degree in the network (D) and was calculated as follows: W = E^*^D. To calculate E, we first defined an interval (I) based on the expression profile of control samples for gene i: I = M-N, where M was calculated as the mean + standard deviation and N was calculated as the mean - standard deviation for gene i. Thus, if the expression (exp) of one gene in one sample did not exceed its I, we considered this gene intended to show a non-cancer expression pattern and E = 0; Otherwise, E = exp-M when exp was more than M and E = exp-N when exp was less than M. The genes with more absolute W were considered as more important, where genes were considered as up-regulated if W was positive and down-regulated if W was negative.

### Calculate pathway scores

For pathway P in each sample, which contains m up-regulated genes and n down-regulated genes, we calculated a score as following:

$$\text{pathwayscore}=\log\frac{\sqrt{\sum_{1}^{m}{\left(d_{i}-\mu_{i}\right)}^{2}}}{\sqrt{\sum_{1}^{n}{\left(d_{j}-\mu_{j}\right)}^{2}}}$$

where *d*_i_ is the expression level of up-regulated gene i in HCC samples and μ_i_ is the mean expression level of gene i in control samples. Similarly, *d*_j_ and μ_j_ represent the expression level of down-regulated gene j in HCC samples and the mean expression level of gene j in control samples, respectively. If the pathwayscore is positive, we consider the pathway shows an active pattern; if the pathwayscore is negative, the pathway is considered to show an inactive pattern.

### Generation of random forest regression model

Random forest is an ensemble method which combines many classification or regression trees [30]. Random forest algorithm has been widely used in the field of bioinformatics, such as transcriptional regulation [31]. Random forest can cope with a large set of correlated variables as well as complex interaction structures and it has already shown excellent performance without any tuning parameters [32]. In random forest, two methods are used to ensure the models to reject randomness: (i) Bootstrap aggregation, that is, each tree in the forest is constructed based on a set of randomly selected samples from the training cases (default to 70% for regression). (ii) Random Subspace Method, that is, a small group of input variables are selected at random, at each node to split (default to 30% for regression). The final decision is generated by majority voting from aggregation of the predictions of all trees.

For the classification model, we used the 433 samples which were initially analyzed as training set and a second set of data which contained 511 samples were used as test set (GSE25097). A forest of 500 trees was fitted to distinguish HCC patients and normal individuals. The other three test sets (GSE49515, GSE64041 and GSE95698) did the same course as above.

The random forest model was constructed using the R package “randomForest” (version: 4.6–12).

### Significance analysis of the model

To validate the significance of our model identified using random forest algorithm, we randomly selected 45 genes from all genes commonly contained by the two data set (the training data set and one test set) and trained a model based on the initial expression profile of 433 samples. Then the model was applied to each test set to distinguish HCC patients and normal individuals. For every test set, this process was repeated for 1000 times and the *P* value was calculated through determining how many times the AUCs were more than the observed one.

## SUPPLEMENTARY MATERIALS TABLES














