# Supplementary material for: Predicting hepatocellular carcinoma through cross-talk genes identified by risk pathways
Source: Oncotarget. 2018 Apr 20;9(30):21259–67. doi: 10.18632/oncotarget.24915 (PMC5940387; doi:10.18632/oncotarget.24915)
Supplement: Supplementary file 1 [file oncotarget-09-21259-s001.pdf]

Predicting hepatocellular carcinoma through cross-talk genes identified by risk pathways

SUPPLEMENTARY MATERIALS

Supplementary Table 1: Differentially expressed genes. See Supplementary_Table_1

Supplementary Table 2: Functions enriched by the genes in HCC-specific network. See Supplementary_Table_2

Supplementary Table 3: The state scores of genes in HCC-specific network. See Supplementary_Table_3

Supplementary Table 4: 104 initial cross-talk genes and their enriched pathways. See Supplementary_Table_4

Supplementary Table 5: 45 cross-talk genes in our model and their enriched pathways. See Supplementary_Table_5

Supplementary Table 6: The comparison of functions enriched by 45 cross-talk genes and others. See Supplementary_Table_6
